# Bayesian data integration for quantifying the contribution of diverse measurements to parameter estimates

**DOI:** 10.1093/bioinformatics/btx666

**Published:** 2017-10-24

**Authors:** Bram Thijssen, Tjeerd M H Dijkstra, Tom Heskes, Lodewyk F A Wessels

**Affiliations:** 1 Computational Cancer Biology, Division of Molecular Carcinogenesis, Netherlands Cancer Institute, CX, Amsterdam, The Netherlands; 2 Department of Protein Evolution, Max Planck Institute for Developmental Biology, Tübingen, Germany; 3 Centre for Integrative Neuroscience, University Clinic Tübingen, Tübingen, Germany; 4 Institute for Computing and Information Sciences, Radboud University Nijmegen, Nijmegen GL, The Netherlands; 5 Faculty of EEMCS, Delft University of Technology, Delft, CD, The Netherlands

## Abstract

**Motivation:**

Computational models in biology are frequently underdetermined, due to limits in our capacity to measure biological systems. In particular, mechanistic models often contain parameters whose values are not constrained by a single type of measurement. It may be possible to achieve better model determination by combining the information contained in different types of measurements. Bayesian statistics provides a convenient framework for this, allowing a quantification of the reduction in uncertainty with each additional measurement type. We wished to explore whether such integration is feasible and whether it can allow computational models to be more accurately determined.

**Results:**

We created an ordinary differential equation model of cell cycle regulation in budding yeast and integrated data from 13 different studies covering different experimental techniques. We found that for some parameters, a single type of measurement, relative time course mRNA expression, is sufficient to constrain them. Other parameters, however, were only constrained when two types of measurements were combined, namely relative time course and absolute transcript concentration. Comparing the estimates to measurements from three additional, independent studies, we found that the degradation and transcription rates indeed matched the model predictions in order of magnitude. The predicted translation rate was incorrect however, thus revealing a deficiency in the model. Since this parameter was not constrained by any of the measurement types separately, it was only possible to falsify the model when integrating multiple types of measurements. In conclusion, this study shows that integrating multiple measurement types can allow models to be more accurately determined.

**Availability and implementation:**

The models and files required for running the inference are included in the Supplementary information.

**Supplementary information:**

Supplementary data are available at *Bioinformatics* online.

## 1 Introduction

Computational models in biology are frequently underdetermined (Gutenkunst *et al.*, 2007), which can limit their usefulness. This underdetermination is a result of our limited capacity to measure biological systems. A dynamic model of an intracellular regulatory network, for example, might contain several proteins of interest that carry out important functions in the system. We would then ideally like to know the concentrations of all these proteins in their various states and complexes, inside the cell, over time. But such direct measurements are currently not possible. Instead we are limited to indirect measurements such as relative protein levels compared to a control, reporter-based measurements, or averages over populations of cells. A compounding difficulty is that the measurements are often relatively noisy. It is thus challenging to accurately determine the unknown parameters of computational models of biological systems.

Intuitively, one would expect that multiple types of measurements, obtained using different experimental techniques, provide more information than a single type of measurement. The combined information would then be more likely to constrain the parameters in a computational model compared to using only a single measurement type. However, this need not be the case; perhaps one particular dataset, such as the most detailed measurements, already contains all relevant information, making additional datasets irrelevant.

The quantification of how much information a dataset brings to the parameter estimates can be achieved using Bayesian statistics (Vyshemirsky and Girolami, 2008; Wilkinson, 2007). For all unknowns in a model, a probability distribution is specified which quantifies the uncertainty in the parameters. This probability distribution can then be updated based on each of the different datasets, using Bayes’ theorem. This provides a convenient framework for the integration of multiple datasets, as it allows a detailed comparison of the amount of information that can be extracted from each of the datasets.

Bayesian statistics has been applied to mechanistic computational models in biology in various settings and model types, including regulatory network models based on ordinary differential equations (ODEs) (Eydgahi *et al.*, 2013; Hug *et al.*, 2013; Toni and Stumpf, 2010; Vyshemirsky and Girolami, 2008; Xu *et al.*, 2010). These applications have so far been limited to the use of a single dataset consisting of one type of measurement. It is thus unclear whether integration of multiple data types within the Bayesian formalism is feasible in practice and whether it is beneficial for achieving more accurate parameter estimates. The purpose of this study was to test the feasibility of this type of data integration and to explore whether multiple data types can indeed provide more accurate parameter estimates.

**Fig. 1.**
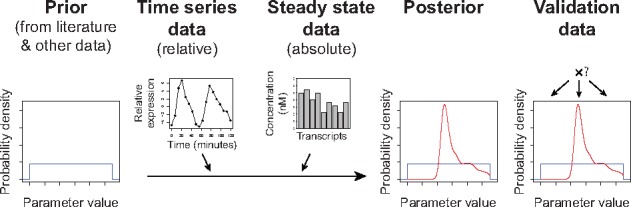
Outline of the approach of data integration using Bayesian statistics. Several initial datasets are assimilated into a prior probability distribution for all parameters in the model. Subsequently, multiple datasets are integrated to update the prior and obtain a posterior probability distribution for all parameters. Finally, this posterior probability distribution is compared to validation data

We tested this approach using a model of a well-studied system, cell cycle regulation in budding yeast. Figure 1 shows the concept of data integration we used: various measurements are included as prior information, subsequently two types of data are incorporated during the inference, and finally the obtained parameter estimates are compared to measurements of these rates from independent studies. 

## 2 Approach and results

### 2.1 Constructing an initial model

**Fig. 2.**
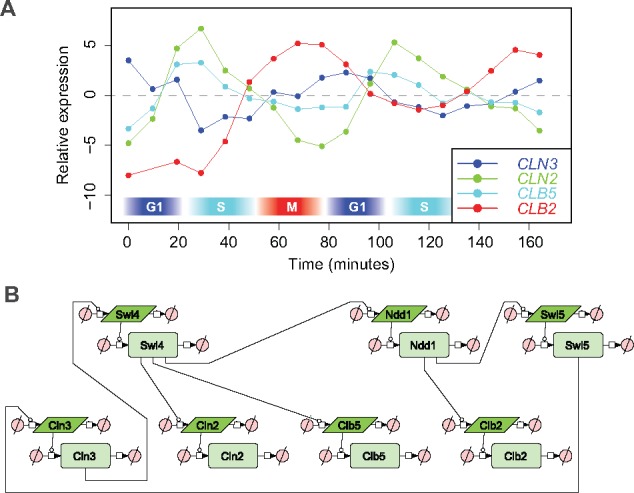
Cyclins and model overview. (**A**) The expression patterns of the four cyclins included in the model. The measurements are from Spellman *et al.* (2003). The approximate cell cycle phase is indicated at the bottom. (**B**) Initial structure of the model in Systems Biology Graphical Notation

We will use cell cycle regulation in budding yeast as test case, as this system is well studied and there is a host of data available. A central event in cell cycle regulation is the cyclic expression of the cyclin proteins (Morgan, 2007). We wished to model the cyclic expression pattern of four cyclins in particular: the G_1_-phase cyclin *CLN3*, the G_1_/S-transition cyclin *CLN2*, the S-phase cyclin *CLB5* and the M-phase cyclin *CLB2* (Fig. 2A). 

**Fig. 3.**
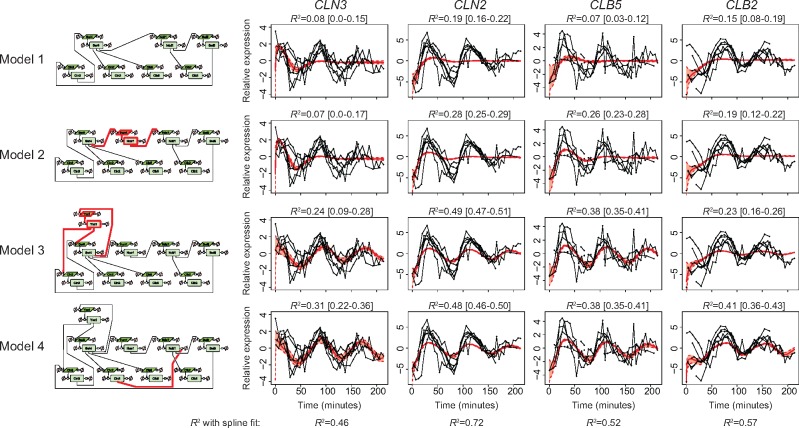
Creating a model that can fit the time course mRNA data. On the left the model structure is indicated in Systems Biology Graphical Notation, with the simplest model at the top. The changes with respect to the previous model are highlighted in red. On the right the mRNA time course measurement data of the cyclins is shown, overlaid with the posterior predictive of the mean of the data. The thick red line indicates the median and the shaded red area indicates the 90% confidence interval. Above each graph the median *R*^2^ is shown and the 90% confidence interval is given in brackets (Color version of this figure is available at *Bioinformatics* online.)

Although many models have been constructed of this system, for example (Chen *et al.*, 2004; Tyson, 1991), we wished to obtain a simple, sparse model that is sufficient for explaining the cyclic expression of the cyclins. To this end we created a simple model that might be able to do this. The structure of this initial model is shown in Figure 2B and the reasoning behind it is as follows. Since the expression of the cyclins oscillate at the transcriptional level, we need to include the transcription factors that are responsible for regulating the transcription of the cyclins in the model. Thus, based on the overview of the cell cycle provided by Morgan (2007), and especially Figure 3-34 therein, we included the three transcription factor complexes SBF, Mcm-Fkh and Swi/Snf. Each of these complexes is represented by one of their subunits: SBF is represented by the regulatory subunit *SWI4*, Mcm1-Fkh is represented by the coactivator *NDD1* and Swi/Snf is represented by the subunit *SWI5*. We chose these subunits because they are regulatory factors and are transcriptionally oscillating (Santos *et al.*, 2015). As most data are available at the mRNA level, we explicitly included the mRNA transcripts as well as the proteins as species in the model. The dynamics are modeled by including rates for transcription, translation and degradation of both mRNA and protein. To keep the model manageable, we did not explicitly include processes such as post-translational modifications, complex formation and intracellular localization. While these processes are also clearly important for cell cycle regulation, the goal is not to create a detailed model but rather a simple model that is sufficient for explaining the cyclic expression of the cyclins. For the same reason, the model contains fewer signaling events than the more comprehensive model of Chen *et al.* (2004). The starting model described here will likely require improvement, which we consider below. Starting from a simple model allows us to find a balance between model complexity and data fit. The resulting model can then be used for testing the integration of multiple datasets. 

**Fig. 4.**
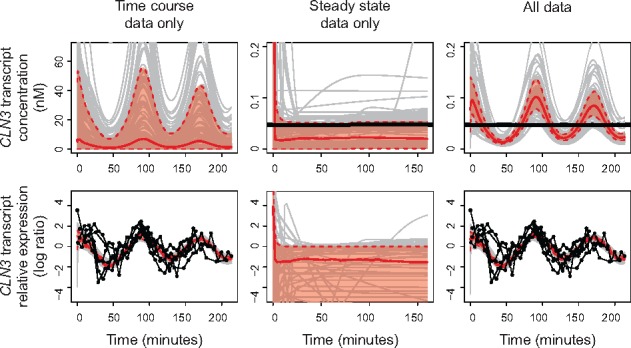
Measurements and posterior predictive with the two types of data separately and together for the CLN3 transcript. The top row shows the absolute concentrations and the bottom row shows the expression relative to the time average (by log ratio). In the left column, only time course data is used, in the middle column only steady-state data is used and in the right column both types of data are used. The measurements are shown in black: horizontal line for absolute steady-state data (note that each line is only a single measurement value), and connected dots for relative time course data (here each dot is a measurement value). The thick red line indicates the median of the posterior predictive and the shaded red area indicates the 90% confidence interval. Grey lines indicate individual trajectories for 100 posterior parameter samples (Color version of this figure is available at *Bioinformatics* online.)

**Fig. 5.**
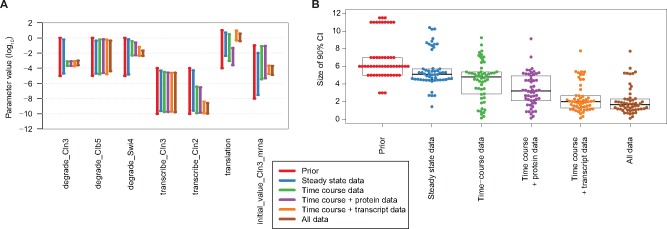
(**A**) Posterior 90% confidence intervals for several model parameters, as inferred using the absolute steady-state data, relative time course data, the time course data together with absolute transcript or protein data, or all data. The confidence intervals and posterior probability densities for all parameters are given in Supplementary Figures S1 and S2. (**B**) Size of the 90% confidence intervals of all parameters on log10 scale. For the prior, the full range is given. Values are given in Supplementary Table S1

**Fig. 6.**
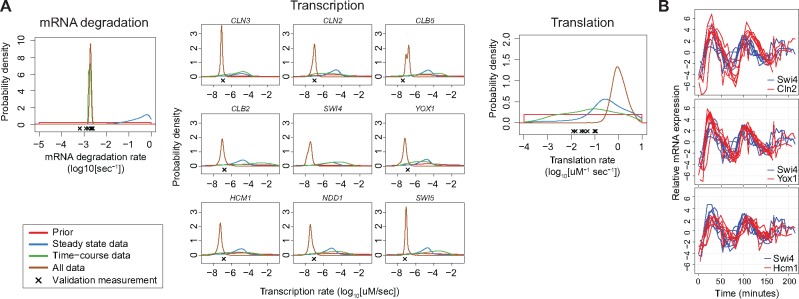
Comparison of parameter estimates with validation data. (**A**) Posterior probability distributions of the parameters, as inferred using the absolute steady-state data, relative time course data or both. The validation measurements are marked with a black cross below the probability distributions. (**B**) Trajectories for the mRNA expression levels of the transcription factor subunit SWI4 and its target genes *CLN2*, *YOX1* and *HCM1*

Another important modeling choice is that we specified the model entirely in physical units of concentration (micromolars) and time (seconds), rather than using dimensionless parameters and abundances. The physical units allow a comparison of the parameter estimates to measurements from independent studies. The model is specified in terms of ODEs, and the rate equations are based on mass action kinetics with the addition of a non-linear term for modeling inhibitory effects. The model is described in more detail in Section 3, and SBML versions of all models are included in Supplementary File S1.

### 2.2 Constructing priors from several datasets

The Bayesian analysis required us to specify prior probabilities for the unknown parameters in the model. For each of the parameters, we specified priors based either on biochemical limits or on published datasets providing information for a parameter. The prior probability distributions and how they were established are described in more detail in the Supplementary Methods. All datasets used throughout this study are listed in Table 1.

**Table 1.** All datasets used in this study

Measurement	Experimental technique	Used as	Reference
Protein concentration	2D-gel electrophoresis	Prior	Futcher *et al.*, 1999
mRNA concentration	Hybridization kinetics	Prior	Hereford and Rosbash, 1977
Cell size	Electrical conductivity	Conversion	Tyson *et al.*, 1979
Transcript elongation rate	ChIP	Prior	Mason and Struhl, 2005
RNA polymerase footprint	Nuclease digestion	Prior	Selby *et al.*, 1997
Peptide elongation rate	Radioactive labeling	Prior	Boehlke and Friesen, 1975; Waldron *et al.*, 1974
Ribosome footprint	Nuclease digestion	Prior	Wolin and Walter, 1988
mRNA time course (relative)	Microarray	Inference	Granovskaia *et al.*, 2010; Pramila *et al.*, 2006; Spellman *et al.*, 2003
mRNA concentration	SAGE	Inference	Velculescu *et al.*, 1997
mRNA concentration	Microarray	Inference	Holstege *et al.*, 1998
Protein concentration	TAP tag; western blot	Inference	Ghaemmaghami *et al.*, 2003
Protein concentration	GFP tag; flow cytometry	Inference	Newman *et al.*, 2006
Protein concentration	2D-HPLC; MS/MS	Inference	Lu *et al.*, 2007
mRNA degradation rate	Microarray	Validation	Wang *et al.*, 2002
Transcription rate	GRO; ChIP-chip	Validation	Pelechano *et al.*, 2011
Translation rate	Polysome profiling	Validation	Arava *et al.*, 2003

### 2.3 Fitting time course mRNA measurement data

As we wished to obtain a model for the cyclic expression of the cyclins, we first turned to measurements of mRNA gene expression over time (Granovskaia *et al.*, 2010; Pramila *et al.*, 2006; Spellman *et al.*, 2003) and tested whether the model can fit these datasets.

A complication with these datasets is that the measurements were taken under different growth conditions, with different synchronization methods and with slightly different yeast strains, resulting in different doubling times for the cells, ranging from 60 to 100 min. To make the datasets compatible, we used the time-normalized data provided by Cyclebase (Santos *et al.*, 2015), and scaled the times back to an 80-min cell cycle, which is a typical doubling time for yeast cells in rich yeast extract peptone dextrose (YEPD) medium (Tyson *et al.*, 1979).

We fitted the model to these three gene expression datasets simultaneously. The measurements are all made on synchronized cells relative to unsynchronized controls, and the likelihood function was specified such that it reflects this. Specifically, the likelihood of the observed values was centered on the log ratio of the modeled transcript concentration divided by the average modeled concentration over time (see Section 3). We expected that the model would not exactly match the measurement data, and so a *t*-distribution was used as error model, such that occasional outlying measurements with respect to the model are not penalized too heavily.

The posterior probabilities were calculated using Markov chain Monte Carlo (MCMC) sampling. The relatively large number of dimensions, with the prior in each dimension spanning many orders of magnitude, makes this a challenging inference task. To be able to run the inference in reasonable time, the Bayesian inference software package BCM was used (Thijssen *et al.*, 2016). The posterior probability distribution contained sub-optimal modes, we therefore used parallel tempering (Geyer, 1991) to have a means of escaping these. MCMC sampling relies on a proposal distribution; a distribution from which new candidate parameter values are drawn. For the efficiency of the sampler it is important that the proposal distribution reflects the shape and scale of the (unknown) posterior distribution. We therefore used automated blocking (Turek *et al.*, 2017) and adaptively scaled the proposal distributions (see Section 3). Traces and autocorrelation plots for the convergence analysis of all models are included in Supplementary File S2.

To test the goodness of fit, we first used graphical posterior predictive checking. The posterior predictive distribution describes a new, predicted dataset given the fitted model. Overlaying this posterior predictive distribution on the observed measurements provides a convenient way of identifying which data can and cannot be explained by the model. Figure 3 (top row) shows the posterior predictive distribution of the mean of the relative transcript levels in the fitted model overlaid on the observed measurements. It is immediately clear that the model cannot adequately explain the expression patterns of the four cyclins. The model can only fit the first peak of *CLN3* expression, but not the subsequent oscillations or the activation of the other cyclins.

To further quantify the goodness of fit, coefficients of determination (*R*^2^) were calculated for the four cyclins (Fig. 3). We compared these values to the *R*^2^ of a spline fit to the data. The spline fit gives a reference *R*^2^ for the optimal fit that can be achieved. The median *R*^2^ for the model fits range from 0.07 to 0.19, whereas a spline fit gives *R*^2^ values ranging from 0.46 to 0.72, again showing that the initial model is insufficient to explain the expression patterns of the cyclins.

### 2.4 Iterative model refinement to create a well-fitting model for the time course mRNA measurement data

As the simplest model could not adequately fit the transcription data, it was necessary to expand the model with additional explanatory factors. We thus searched the literature to find important mechanisms that were missing from the model. For each addition, we re-fitted the model to the data and compared the posterior predictive distributions and *R*^2^ values for expression of the four cyclins. Note that we could not use the marginal likelihood for model selection here, because when we added additional species to the model we also included the expression data for those new species in the likelihood function. This affects the marginal likelihood; the marginal likelihood of two differing sets of data cannot be compared to each other.

The first addition to the model which we considered was the transcription factor *HCM1*. There is a significant delay between the transcriptional peak of *SWI4* and *NDD1*, especially compared to the peaks of *CLN2* and *CLB5*, which occur more rapidly after the expression of *SWI4* (see Supplementary File S2 for the trajectories of all species). The transcription factor *HCM1* has been found to be an important part of the transcriptional cell cycle regulation system (Pramila *et al.*, 2006), and the inclusion of this factor could introduce the necessary delay in the model. As shown in Figure 3 (second row), the addition of *HCM1* indeed improved the fit of the model, particularly for the induction of the expression of *CLN2* and *CLB5* after *SWI4* expression. However, the model was still not able to explain the oscillatory aspect of the expression of the four cyclins.

The lack of oscillatory behavior of the model suggested that a feedback loop might be required. We therefore considered the addition of the inhibitory transcription factor *YOX1* (Pramila *et al.*, 2002). This transcription factor provides a negative feedback loop from *SWI4* back to both *SWI4* and *CLN3*. As shown in Figure 3 (third row), with this addition the model could indeed recapitulate the oscillatory aspect of the expression pattern of the four cyclins.

As the magnitude of the oscillations in *CLB2* was still greater in the data than could be explained by the model, we considered the addition of another regulatory mechanism, namely the degradation of *NDD1* by the anaphase promoting complex (Sajman *et al.*, 2015). This complex is normally active, unless it is inactivated by *CLN2* (Morgan, 2007). Thus, *NDD1* would be actively degraded until *CLN2* signals the start of S-phase. As shown in Figure 3 (bottom row), with the addition of this mechanism the model can indeed better explain the expression pattern of the *NDD1*-target *CLB2*.

With these additions to the model, the expression patterns of the four cyclins are adequately explained (*R*^2^ > 0.3 for all cyclins, and at least 65% of the *R*^2^ achieved with a spline fit). Although further additions can be considered, we wished to keep the model as small as possible while achieving a reasonable fit. This was mainly done to keep the computational requirements manageable—to generate 1000 posterior samples for the fourth extended model required approximately 60 h. The structure of the resulting model is similar to the Boolean network model of Orlando *et al.* (2008) in terms of the transcriptional regulatory network.

### 2.5 Simultaneous fitting of time course and steady-state measurement data

Now that the model is able to explain the relative time course measurements adequately, we can start including additional datasets to test whether the parameters of the model can be more tightly constrained with the integration of additional data. We turned to absolute measurements of the mRNA (Holstege *et al.*, 1998; Velculescu *et al.*, 1997) and protein (Ghaemmaghami *et al.*, 2003; Lu *et al.*, 2007; Newman *et al.*, 2006) concentrations of the species in the model. These measurements were done at steady-state growth conditions in non-synchronized cells. We incorporated this in the likelihood by taking the time average of the modeled trajectories, and setting this time average as the modeled value of the steady-state data, where the time average was taken over two cell cycles.

The addition of absolute concentration data to relative time series data may seem trivial, and it could potentially also be achieved by transforming the kinetic parameters and concentrations accordingly. However, keeping the model specified in physical dimensions (micromolars and seconds) is natural, and more importantly, it allows for a direct comparison of the kinetic rates with measurements of these rates later on.

Figure 4 shows the posterior trajectories of the transcripts of one of the cyclins, *CLN3*, after fitting the relative time course data alone, the absolute steady-state data alone or all data together (trajectories for all other species in the model are included in Supplementary File S2). Several observations can be made. First, it is apparent that the absolute concentrations can be quite high when only time course data is used. When the steady-state data is included however, the concentrations are constrained to be much lower. Second, when only steady-state data is used, the model displays various behaviors including stable expression, decay and oscillations (see the individual trajectories depicted in grey)—each of these behaviors would be consistent with the given average data over a period of two cell cycles. With all measurements types included, the model displays the correct oscillations at concentrations consistent with the steady-state data. Finally, the fit to the relative time course data is not compromised by the inclusion of the absolute steady-state data, and vice versa. The model is thus able to fit both types of data at the same time, and no modifications need to be made to the model structure to accommodate the steady-state data.

Figure 5A shows the 90% posterior confidence intervals for several parameters in the model, for the relative time course data alone, the absolute steady-state data alone or all data together (confidence intervals and density plots for all parameters are included in Supplementary Figs S1 and S2). For several parameters, each data type separately provides some information, but the inclusion of the two types of data together provides significantly tighter confidence intervals, for example for the translation rate. There are also parameters that can already be inferred from the time course data alone; that is, for these parameters the addition of the steady-state data does not reduce the confidence intervals, such as the degradation rate of *CLN3*. In many cases, the steady-state data by itself provides little information for constraining the parameters, which is not surprising for a dynamic model. However, the addition of the steady-state data to the time course data does reduce the uncertainty compared to the time course data alone. Examples of this are the degradation rate of *SWI4* or the transcription rate of *CLN2.*

In general across all parameters, combining multiple types of measurements reduces the uncertainty in parameter estimates (Fig. 5B). With all data types included, 45 out of 54 parameters have 90% confidence intervals of less than half of the prior range, whereas the steady-state data by itself constrains only 1 parameter to this extent and the time course data 14 parameters. Comparing the added value of the absolute protein and transcript concentrations, we note that it is mainly the transcript concentrations that reduces the uncertainty (columns 4 and 5 in Fig. 5B and see Supplementary Table S1 for the values). Nevertheless, adding the protein concentration data to the time course and transcript concentration data still further reduces the uncertainty for several parameters.

### 2.6 Comparison of parameter estimates with rate measurement data

To test whether the obtained parameter estimates are accurate, we compared them to measurements from three additional, independent datasets. In particular, the mRNA degradation rate (Wang *et al.*, 2002), transcription rates (Pelechano *et al.*, 2011) and translation rates (Arava *et al.*, 2003) have been measured for budding yeast. Figure 6A shows the measured values of the parameters compared to the posterior probability distributions of the parameter estimates.

For the mRNA degradation rate, the measurements are in close agreement with the predicted rates (Fig. 6A, left panel). We assumed a common rate parameter for all species, while the measurements were done for each gene separately, and there is indeed some variability between the measurements for the genes that were included in the model. Nevertheless, the measured degradation rates of all genes are within the same order of magnitude as the estimated average degradation rate (the difference between the measurements and the maximum *a posteriori* estimate on log_10_ scale is <0.5), so the scale of the average mRNA degradation rate was predicted accurately.

For the transcription rates, these rates in the model are split into two parts: basal transcription and transcription factor induced transcription. The rate measurements are population averages, and as each cell would be in a different stage of the cell cycle, they will be expressing different levels of the transcription factors. To be able to compare the measurements of the transcription rates to the model’s estimated rates, it is necessary to calculate the total, average transcription rate. This was obtained by averaging the transcription rate of each gene over time. This rate thus includes the effect of the time-varying expression of the transcription factors. When only time course data was used, the transcription rates were not constrained, but they do have non-zero probability at the measured values. However, when all data are included, the estimated transcription rates closely match the measured values for most genes (Fig. 6A, middle panels; seven of the eight measured values lies within the 90% confidence interval, and the remaining gene is at least within the same order of magnitude). Thus, for the transcription rates, the addition of the absolute concentrations to the relative dynamic data constrained the parameter estimates to values close to or matching the measurements of these rates.

The measured translation rates have been estimated from ribosome densities using polysome profiling, whereby a processing speed of 10 amino acids/s was assumed (Arava *et al.*, 2003). Note that the authors mention that their estimates should be used with caution. Nevertheless, assuming the estimates from Arava *et al.* are accurate, then our model estimate using all inference data are two orders of magnitude too high (Fig. 6A, right panel). The model estimate is indeed quite high at around 1 protein/transcript/s. While this is feasible given the prior information, it would require that the transcripts are always essentially fully packed with ribosomes.

To find the reason for this high translation rate estimate, we investigated the trajectories of the transcription factors and their target genes. If we compare the mRNA expression trajectories for the transcription factor *SWI4* and its targets *CLN2*, *HCM1* and *YOX1* (see Fig. 6B), it makes sense that the model requires a high translation rate. The peaks in transcription of the target genes follow very closely after the peak in transcription of the transcription factor, especially in the first cell cycle. Given that this process of rapid induction of transcription in the model has to occur through the translation of the transcription factor, then there are two ways in which the model might fit the data: either the translation rate must be high, or the concentration of the transcript of the transcription factor must be high. When using only the relative data, it is not possible to distinguish between these scenarios; indeed in this case the translation rate is not constrained: the 90% confidence interval of the translation rate spans almost three orders of magnitude (Fig. 5). However, when including both relative and absolute data, the inference can make use of the information that the concentration of the transcription factor is low. It can thus be inferred that the translation rate must be high, given this model.

It is known that other mechanisms are at play here as well, such as the regulation of *SWI4* and the SBF transcription factor complex through phosphorylation by different cyclin/CDKs (Siegmund and Nasmyth, 1996). Indeed it has been shown that induction of G_1_-phase transcripts can occur in the absence of protein translation (Marini and Reed, 1992). It is likely that a model with additional layers of *SWI4* regulation would be able to fit all data with lower translation rates. Unfortunately we were not able to expand the model with such additional effects, as the parameter inference for these expanded models would involve a prohibitive amount of computation time. Regardless, these results show that the model can be identified as being incomplete by using the inference of parameters from multiple datasets. This model deficiency could not be deduced from any of the datasets alone.

## 3 Materials and methods

### 3.1 Model equations

The computational model consists of two types of species: the proteins and the mRNA transcripts. The rate equations for these species are based on mass action kinetics, with the addition of a non-linear term for modeling inhibitory effects. For transcripts, the rate equation contains three terms: one for transcription, one for inhibition of transcription and one for degradation. The transcription rate is proportional to the concentration of the activating transcription factor for that gene. This transcription can be inhibited by an inhibitory transcription factor. Each transcript has exactly one activating transcription factor and at most one inhibitory transcription factor. For proteins, the rate equation also contains three terms: one for translation, one for degradation and one for inhibition of degradation. The translation rate is proportional to the concentration of the transcript for that protein, and the degradation rate is proportional to the concentration of the protein itself. See the Supplementary Methods for a more detailed description and the equations.

### 3.2 Prior distributions

For all parameters, we used uniform priors on a log_10_ scale. A log scale was chosen as we were interested in the orders of magnitude of the parameters rather than their precise values. The limits of the uniform distributions were chosen based on various data points and biochemical limits as described in the Supplementary Methods.

### 3.3 Likelihood

Firstly, the time average of the concentration of a transcript was calculated by averaging over two full cell cycles. Then, for relative time course data measured using synchronized cells relative to unsynchronized cells, we modeled the relative value by dividing the modeled concentration by the time average and taking the log. As error model we used a *t*-distribution with three degrees of freedom, as a means of robust inference (Gelman *et al.*, 2014). This distribution was centered on the log ratio of the relative expression. For the absolute concentration data, the time average value is log_10_ transformed, and again a *t*-distribution is used as error model. As for the prior, the likelihood is specified on a log scale as it is sufficient if the model captures the right order of magnitude. See the Supplementary Methods for the equations.

### 3.4 Model inference

The posterior probability distributions were calculated using parallel-tempered MCMC (Geyer, 1991), using the Bayesian inference software package BCM (Thijssen *et al.*, 2016). For the initial model, we used 32 parallel chains with the temperatures of the chains distributed quadratically. The burn-in period was set to 1.25 million samples followed by a sampling period of 5 million posterior samples, which were subsampled at 1 in 2500. At each step, a random choice was made between updating each chain with five Metropolis–Hastings steps and swapping a random adjacent pair of chains. The probability of selecting a swap step was set to 0.99. For the proposal distribution in the Metropolis–Hastings steps, the parameters were blocked automatically (Turek *et al.*, 2017) and we used a multivariate normal distribution for each block of parameters. The proposal covariance matrix for each block was set to the empirical covariance of the preceding samples and adaptively scaled to obtain an acceptance rate of 0.23 within each block. These settings produced sufficiently uncorrelated posterior samples (see Supplementary File S2 for traces and autocorrelation plots) and were sufficient to achieve at least 100 round trips from prior to posterior. The sampling period and subsampling was doubled for model 3 and quadrupled for model 4, such that the resulting posterior samples were still sufficiently uncorrelated and at least 100 round trips from prior to posterior were achieved.

All files required for running the inference in BCM, including the prior and likelihood specification, the models in SBML/CellDesigner format, as well as a NetCDF archive containing the pre-processed data, are included in Supplementary File S1.

### 3.5 Model checking

The model fit was investigated using the posterior predictive distribution and coefficients of determination. The posterior predictive distribution is the probability distribution of a new set of data, given the model and the observed data. This distribution was approximated using the posterior Monte Carlo samples. The coefficients of determination for the time course data were calculated relative to a null model, which has a separate mean for each experiment. A reference *R*^2^ was calculated by fitting a cubic spline to the data with the smoothing parameter selected through cross-validation. See the Supplementary Methods for details and equations.

## 4 Discussion

Model determination is an important aspect of computational modeling. Models in systems biology are frequently underdetermined, and as a result it is often not possible to confirm or falsify a particular model. There is thus a need for methods to determine models more accurately. With the increasing amount of data available for many biological systems, the use of multiple datasets to constrain the parameters from different angles is a promising avenue. Bayesian statistics provides a coherent and convenient framework to accomplish this. Here, we have shown that it is feasible to integrate diverse datasets during the Bayesian inference of parameters of an ODE-based model. The process as described here may be useful as a general recipe for integrating diverse measurement types also in other settings. More importantly, we have shown that this integration of diverse data types can provide tighter posterior estimates, at least in obtaining the right order of magnitude, thus achieving more accurate model determination. We noticed that even when a single dataset, taken by itself, provides little information, it can still significantly improve parameter estimates when used in conjunction with other datasets.

There are several challenges when using this type of data integration based on model simulation and Bayesian statistics. The biggest challenge is the scaling of the computational demands with respect to the size of the model. This is due to two reasons. First, the simulation of a computational model typically does not scale well with model size (cubically in the case of direct, implicit ODE solvers). Second, the parameter inference is increasingly challenging when the number of parameters increases. Although in theory Monte Carlo methods scale independently of the dimensionality, this requires that the samples are concentrated in regions of high posterior probability. The efficiency of generating a good set of samples critically depends on the proposal distribution that is used. Given the complex shape of the posterior probability distributions of biological computational models, in particular the presence of multiple modes and ridges (Girolami, 2008; Hug *et al.*, 2013), proposal distributions typically become much less efficient with higher dimensionality. Both of these computational challenges apply more generally to any approach using model simulation and global parameter inference. Increases in computational capabilities, more efficient simulation methods and sampling or optimization methods tailored for the inference of biological computational models may allow larger models in the future.

For budding yeast, and their cell cycle regulation in particular, many more measurements have been performed, such as mRNA quantification by qPCR (Miura *et al.*, 2008) and RNA sequencing (Nagalakshmi *et al.*, 2008), protein-level time course data by mass spectrometry (Flory *et al.*, 2006) and GFP-tagged time lapse microscopy (Ball *et al.*, 2011). In principle, these data can be integrated with the same approach as was done for the data in this study, and it would be interesting to explore the contributions and concordance of these measurements. A challenge for further integration of time course data is the synchronization of the timing, which is not straightforward when using different experimental setups. This synchronization can also directly affect kinetic rates, for example the alignment of transcript and protein time course data can directly influence the estimated translation rate.

To be able to compare the contribution of the different datatypes, it is necessary to quantify the uncertainty in the parameter estimates, which was achieved here using Bayesian statistics. The quantification of uncertainty has previously been achieved with different approaches as well (reviewed by Vanlier *et al.*, 2013), including using the profile likelihood (Raue *et al.*, 2009) and through bootstrapping (Brännmark *et al.*, 2010). The incorporation of multiple datasets in the likelihood function can in principle be translated to these formalisms as well. A unique advantage of the Bayesian approach is the ability to explicitly include data as prior information, which we have utilized to incorporate various datasets. Profile likelihoods may be computationally more efficient to calculate than posterior probabilities, although the calculations still involve the most challenging aspect, namely global optimization. The profile likelihood is limited in that it provides uncertainty estimates for each parameter separately rather than for all parameters jointly.

In conclusion, we have shown that diverse types of measurements can be successfully integrated during the inference of parameters of ODE systems using Bayesian statistics. This integration provided more tightly constrained parameter estimates, thereby achieving a more accurate model determination.

## Funding

This work was performed within the Cancer Genomics Netherlands Program supported by the Gravitation program of the Netherlands Organization for Scientific Research (NWO). This work was sponsored by NWO Physical Sciences for the use of supercomputer facilities. This work was further supported by CogIMon H2020 ICT-644727 to T.D from the European Commission.

*Conflict of Interest*: none declared.

## Supplementary Material

Supplementary Data

## References

[btx666-B1] Arava Y. et al (2003) Genome-wide analysis of mRNA translation profiles in *Saccharomyces cerevisiae*. Proc. Natl. Acad. Sci. USA, 100, 3889–3894. 12660367 10.1073/pnas.0635171100 PMC153018

[btx666-B2] Ball D.A. et al (2011) Oscillatory dynamics of cell cycle proteins in single yeast cells analyzed by imaging cytometry. PLoS One, 6. 10.1371/journal.pone.0026272 PMC3202528 22046265

[btx666-B3] Boehlke K.W., Friesen J.D. (1975) Cellular content of ribonucleic acid and protein in *Saccharomyces cerevisiae* as a function of exponential growth rate: calculation of the apparent peptide chain elongation rate. J. Bacteriol., 121, 429–433. 1089627 10.1128/jb.121.2.429-433.1975 PMC245948

[btx666-B4] Brännmark C. et al (2010) Mass and information feedbacks through receptor endocytosis govern insulin signaling as revealed using a parameter-free modeling framework. J. Biol. Chem., 285, 20171–20179. 20421297 10.1074/jbc.M110.106849 PMC2888430

[btx666-B5] Chen K.C. et al (2004) Integrative analysis of cell cycle control in budding yeast. Mol. Biol. Cell, 15, 3841–3862. 15169868 10.1091/mbc.E03-11-0794 PMC491841

[btx666-B6] Eydgahi H. et al (2013) Properties of cell death models calibrated and compared using Bayesian approaches. Mol. Syst. Biol., 9, 644. 23385484 10.1038/msb.2012.69 PMC3588908

[btx666-B7] Flory M.R. et al (2006) Quantitative proteomic analysis of the budding yeast cell cycle using acid-cleavable isotope-coded affinity tag reagents. Proteomics, 6, 6146–6157. 17133367 10.1002/pmic.200600159

[btx666-B8] Futcher B. et al (1999) A sampling of the yeast proteome. Mol. Cell. Biol., 19, 7357–7368. 10523624 10.1128/mcb.19.11.7357 PMC84729

[btx666-B9] Gelman A. et al (2014) Models for robust inference, In: Bayesian Data Analysis. 3rd edn, Chapman & Hall/CRC, Boca Raton, FL, pp. 435–446.

[btx666-B10] Geyer C.J. (1991) Markov Chain Monte Carlo maximum likelihood. Computing Science and Statistics: Proceedings of the 23rd Symposium Interface 156–163.

[btx666-B11] Ghaemmaghami S. et al (2003) Global analysis of protein expression in yeast. Nature, 425, 737–741. 14562106 10.1038/nature02046

[btx666-B12] Girolami M. (2008) Bayesian inference for differential equations. Theor. Comput. Sci., 408, 4–16.

[btx666-B13] Granovskaia M.V. et al (2010) High-resolution transcription atlas of the mitotic cell cycle in budding yeast. Genome Biol., 11, R24. 20193063 10.1186/gb-2010-11-3-r24 PMC2864564

[btx666-B14] Gutenkunst R.N. et al (2007) Universally sloppy parameter sensitivities in systems biology models. PLoS Comput. Biol., 3, 1871–1878. 17922568 10.1371/journal.pcbi.0030189 PMC2000971

[btx666-B15] Hereford L.M., Rosbash M. (1977) Number and distribution of polyadenylated RNA sequences in yeast. Cell, 10, 453–462. 321129 10.1016/0092-8674(77)90032-0

[btx666-B16] Holstege F.C. et al (1998) Dissecting the regulatory circuitry of a eukaryotic genome. Cell, 95, 717–728. 9845373 10.1016/s0092-8674(00)81641-4

[btx666-B17] Hug S. et al (2013) High-dimensional Bayesian parameter estimation: case study for a model of JAK2/STAT5 signaling. Math. Biosci., 246, 293–304. 23602931 10.1016/j.mbs.2013.04.002

[btx666-B18] Lu P. et al (2007) Absolute protein expression profiling estimates the relative contributions of transcriptional and translational regulation. Nat. Biotechnol., 25, 117–124. 17187058 10.1038/nbt1270

[btx666-B19] Marini N.J., Reed S.I. (1992) Direct induction of G1-specific transcripts following reactivation of the Cdc28 kinase in the absence of *de novo* protein synthesis. Genes Dev., 6, 557–567. 1313770 10.1101/gad.6.4.557

[btx666-B20] Mason P.B., Struhl K. (2005) Distinction and relationship between elongation rate and processivity of RNA polymerase II *in vivo*. Mol. Cell, 17, 831–840. 15780939 10.1016/j.molcel.2005.02.017

[btx666-B21] Miura F. et al (2008) Absolute quantification of the budding yeast transcriptome by means of competitive PCR between genomic and complementary DNAs. BMC Genomics, 9, 574. 19040753 10.1186/1471-2164-9-574 PMC2612024

[btx666-B22] Morgan D.O. (2007) The Cell Cycle: Principles of Control. Corby, UK: Oxford University Press.

[btx666-B23] Nagalakshmi U. et al (2008) The transcriptional landscape of the yeast genome defined by RNA sequencing. Science, 320, 1344–1349. 18451266 10.1126/science.1158441 PMC2951732

[btx666-B24] Newman J.R.S. et al (2006) Single-cell proteomic analysis of *S. cerevisiae* reveals the architecture of biological noise. Nature, 441, 840–846. 16699522 10.1038/nature04785

[btx666-B25] Orlando D.A. et al (2008) Global control of cell-cycle transcription by coupled CDK and network oscillators. Nature, 453, 944–947. 18463633 10.1038/nature06955 PMC2736871

[btx666-B26] Pelechano V. et al (2011) A complete set of nascent transcription rates for yeast genes. Curr. Sci., 101, 1435–1439. 10.1371/journal.pone.0015442 PMC2982843 21103382

[btx666-B27] Pramila T. et al (2002) Conserved homeodomain proteins interact with MADS box protein Mcm1 to restrict ECB-dependent transcription to the M/G1 phase of the cell cycle. Genes Dev., 16, 3034–3045. 12464633 10.1101/gad.1034302 PMC187489

[btx666-B28] Pramila T. et al (2006) The Forkhead transcription factor Hcm1 regulates chromosome segregation genes and fills the S-phase gap in the transcriptional circuitry of the cell cycle. Genes Dev., 20, 2266–2278. 16912276 10.1101/gad.1450606 PMC1553209

[btx666-B29] Raue A. et al (2009) Structural and practical identifiability analysis of partially observed dynamical models by exploiting the profile likelihood. Bioinformatics, 25, 1923–1929. 19505944 10.1093/bioinformatics/btp358

[btx666-B30] Sajman J. et al (2015) Degradation of Ndd1 by APC/CCdh1 generates a feed forward loop that times mitotic protein accumulation. Nat. Commun., 6, 7075. 10.1038/ncomms8075 25959309

[btx666-B31] Santos A. et al (2015) Cyclebase 3.0: a multi-organism database on cell-cycle regulation and phenotypes. Nucleic Acids Res., 43, D1140–D1144. 25378319 10.1093/nar/gku1092 PMC4383920

[btx666-B32] Selby C.P. et al (1997) RNA polymerase II stalled at a thymine dimer: footprint and effect on excision repair. Nucleic Acids Res., 25, 787–793. 9016630 10.1093/nar/25.4.787 PMC146523

[btx666-B33] Siegmund R.F., Nasmyth K.A. (1996) The *Saccharomyces cerevisiae* start-specific transcription factor Swi4 interacts through the ankyrin repeats with the mitotic Clb2/Cdc28 kinase and through its conserved carboxy terminus with Swi6. Mol. Cell. Biol., 16, 2647–2655. 8649372 10.1128/mcb.16.6.2647 PMC231255

[btx666-B34] Spellman P.T. et al (2003) Comprehensive identification of cell cycle-regulated genes of the yeast *Saccharomyces cerevisiae* by microarray hybridization. Mol. Biol. Cell, 9, 3273–3297. 10.1091/mbc.9.12.3273 PMC25624 9843569

[btx666-B35] Thijssen B. et al (2016) BCM: toolkit for Bayesian analysis of computational models using samplers. BMC Syst. Biol., 10, 100. 27769238 10.1186/s12918-016-0339-3 PMC5073811

[btx666-B36] Toni T., Stumpf M.P.H. (2010) Simulation-based model selection for dynamical systems in systems and population biology. Bioinformatics, 26, 104–110. 19880371 10.1093/bioinformatics/btp619 PMC2796821

[btx666-B37] Turek D. et al (2017) Automated Parameter Blocking for Efficient Markov Chain Monte Carlo Sampling. Bayesian Anal., 12, 465–490.

[btx666-B38] Tyson C.B. et al (1979) Dependency of size of *Saccharomyces cerevisiae* cells on growth rate. J. Bacteriol., 138, 92–98. 374379 10.1128/jb.138.1.92-98.1979 PMC218242

[btx666-B39] Tyson J.J. (1991) Modeling the cell division cycle: cdc2 and cyclin interactions. Proc. Natl. Acad. Sci. USA, 88, 7328–7332. 1831270 10.1073/pnas.88.16.7328 PMC52288

[btx666-B40] Vanlier J. et al (2013) Parameter uncertainty in biochemical models described by ordinary differential equations. Math. Biosci., 246, 305–314. 23535194 10.1016/j.mbs.2013.03.006

[btx666-B41] Velculescu V.E. et al (1997) Characterization of the yeast transcriptome. Cell, 88, 243–251. 9008165 10.1016/s0092-8674(00)81845-0

[btx666-B42] Vyshemirsky V., Girolami M. (2008) Bayesian ranking of biochemical system models. Bioinformatics, 24, 833–839. 18057018 10.1093/bioinformatics/btm607

[btx666-B43] Waldron C. et al (1974) The elongation rate of proteins of different molecular weight classes in yeast. FEBS Lett., 46, 11–16. 4607959 10.1016/0014-5793(74)80323-6

[btx666-B44] Wang Y. et al (2002) Precision and functional specificity in mRNA decay. Proc. Natl. Acad. Sci. USA, 99, 5860–5865. 11972065 10.1073/pnas.092538799 PMC122867

[btx666-B45] Wilkinson D.J. (2007) Bayesian methods in bioinformatics and computational systems biology. Brief. Bioinform., 8, 109–116. 17430978 10.1093/bib/bbm007

[btx666-B46] Wolin S.L., Walter P. (1988) Ribosome pausing and stacking during translation of a eukaryotic mRNA. EMBO J., 7, 3559–3569. 2850168 10.1002/j.1460-2075.1988.tb03233.x PMC454858

[btx666-B47] Xu T.-R. et al (2010) Inferring signaling pathway topologies from multiple perturbation measurements of specific biochemical species. Sci. Signal., 3, ra20. 20731071

