# Supplementary material for: Bayesian data integration for quantifying the contribution of diverse measurements to parameter estimates
Source: Bioinformatics. 2017 Oct 24;34(5):803–11. doi: 10.1093/bioinformatics/btx666 (PMC6192208; doi:10.1093/bioinformatics/btx666)
Supplement: Supplementary Data [file btx666_supp.zip › btx666-suppl_data/supplementary information.docx]

## Supplementary Methods

### Model equations

The computational model consists of two types of species: the proteins *p_i_*, and the mRNA transcripts *m_i_*. The rate equations for these species are based on mass action kinetics, with the addition of a nonlinear term for modeling inhibitory effects.

For transcripts, the rate equation contains three terms: one for transcription, one for inhibition of transcription and one for degradation. The three terms are marked in the equation below, and they correspond to the following:

- *transcription*: The transcription rate is a sum of a constant rate and a rate that is proportional to the concentration of the activating transcription factor for that gene. Each transcript has exactly one activating transcription factor.
- *transcription inhibition*: The transcription can be inhibited by an inhibitory transcription factor, which is modeled with a two-parameter logistic function (including a 50% inhibitory concentration and a steepness parameter). Each transcript has at most one inhibitory transcription factor.
- *degradation*: Degradation is modeled as exponential decay.

The rate equation for transcripts is then defined as:

$\frac{dm_{i}}{dt}=\underbrace{\begin{matrix} \\ \begin{matrix} \\ \end{matrix} \end{matrix}(r_{base}+r_{induced,i,j}p_{j})}_{\text{transcription}}\underbrace{\begin{matrix} \\ \begin{matrix} \\ \end{matrix} \end{matrix}\frac{1}{1+e^{s_{k,i}(\log p_{k}-\log c_{k,i})}}}_{\text{transcription inhibition}}-\underbrace{\begin{matrix} \\ \begin{matrix} \\ \end{matrix} \end{matrix}d_{mRNA}\cdot m_{i}}_{\text{degradation}}$ ,

where *m_i_* is the concentration of mRNA transcript *i* in µM, *r_base_* is the base transcription rate in µM/s, *r_induced,I,j_* is the transcription rate of transcript *i* induced by transcription factor *j* in µM/µM/s, *p_j_* is the concentration of the transcription factor *j* in µM, and dmRNA is the degradation rate of all mRNA transcripts in s^-1^. If an inhibitory transcription factor for gene *i* is included in the model, then this inhibitory transcription factor is indicated by index *k*, and *s_k,i_* is the steepness of the inhibition curve and *c_k,i_* is the 50%-inhibitory concentration in µM. If no inhibitory transcription factor is present for gene *i*, the transcription inhibition term is set to 1, such that the transcription is not inhibited.

For proteins, the rate equation also contains three terms: one for translation, one for degradation, and one for inhibition of degradation.

- *translation*: The translation rate is proportional to the concentration of the transcript (*m_i_*) for that protein.
- *degradation*: The degradation is modeled as exponential decay, but it is split into two parts: a first part (*d_i_*) which is constant and represents general decay/degradation of the protein, and a second part (*d_induced,k,i_*) which represents active, specific degradation, which can be inhibited by another protein. Both parts of the degradation rate are proportional to the concentration of the protein itself.
- *Inhibited degradation:* The inhibition of degradation is modeled with a two-parameter logistic function (including a 50% inhibitory concentration and a steepness parameter). Each protein has at most one protein that can inhibit its degradation.

The rate equation for proteins is then defined as:

$\frac{dp_{i}}{dt}=\underbrace{\begin{matrix} \\ \begin{matrix} \\ \end{matrix} \end{matrix}um_{i}}_{\text{translation}}-\underbrace{\begin{matrix} \\ \begin{matrix} \\ \end{matrix} \end{matrix}d_{i}p_{i}}_{\text{degradation}}-\underbrace{\begin{matrix} \\ \begin{matrix} \\ \end{matrix} \end{matrix}\frac{d_{induced,k,i}}{1+e^{s_{k,j}(\log p_{k}-\log c_{k,i})}}p_{i}}_{\text{inhibited degradation}}$ ,

where *p_i_* is the concentration of protein *i* in µM, *u* is the translation rate in µM/µM/s, *m_i_* is the concentration of mRNA transcript *i* in µM, and *d_i_* is the degradation rate of protein *i* in s^-1^. If a degradation-inhibiting protein is included in the model, then this protein is indicated by index *k*, and *d_induced,k,i_* is the degradation rate of protein *i* that can be inhibited by protein *k*, *s_k,i_* is the steepness of the inhibition curve and *c_k,i_* is the 50%-inhibitory concentration in µM. If no degradation-inhibiting protein is present for protein *i*, the inhibited degradation term is set to 0.

### Prior distributions

#### Cell size

For various calculations and conversions, we need the cell size. Although the cell size varies between conditions and during the cell cycle, we assumed that the cell size is 37 μm^3^ and constant. This is an average size for yeast cells growing in rich (YEPD) medium (Tyson and Lord, 1979). Combined with Avogadro’s constant, this means that 1 molecule per cell corresponds to approximately 4.5∙10^-5^ μM.

#### Concentrations and initial conditions

For setting a prior on the initial conditions of proteins, we used the dataset of Futcher *et al* (Futcher *et al.*, 1999). They sampled the yeast proteome and established protein copy number per cell for these proteins. Since the sampling across the proteome in this study was not uniform, but instead focused on the most abundant proteins, we used this dataset only to provide a reasonable upper limit. The most highly expressed proteins were present in the range of 1 million copies per cell, or approximately 45 μM. To provide some margin of error, we set the upper limit of protein concentrations at 100 μM. We assumed 0.1 nM as lower limit (approximately 2.2 molecules per cell).

For setting a prior on the initial conditions of transcripts, we used the measurements of Hereford and Rosbash (Hereford and Rosbash, 1977). They estimated the transcript copy number per cell to vary between 1 and 200 copies per cell. To provide some margin of error, we set the upper limit of transcript concentration at 0.1 μM (approximately 2,200 transcripts per cell). We assumed 10^-8^ μM as lower limit, to allow transcripts to be practically absent as well (<<1 transcript per cell).

#### Transcription rates

The model contains two classes of transcription rate parameters: basal transcription and transcription factor-induced transcription. To allow either of the two types of transcription to be practically absent, we set the lower limit to 10^-10^ μM/s and 10^-10^ μM/μM/s respectively. For the upper limit, we consider the case where transcription initiation is not rate limiting; the transcription rate is then bound by the transcript elongation rate and the number of polymerases transcribing the gene. We assume that the elongation rate is constant; this rate has been estimated at 2 kb/min (Mason and Struhl, 2005). The footprint of RNA polymerase has been estimated at 40 nucleotides (Selby *et al.*, 1997). If a gene is fully packed with polymerases, this gives a transcription rate of approximately 0.8 transcripts/s. To allow for some margin of error, we take the upper limit as 10^-4^ μM/s (approximately 2.2 transcripts/s).

Recall that a priori we expect that proteins are in the concentration range of 0.1 nM to 100 μM. To allow for transcription factors at the lower concentration limit, 0.1 nM, to already fully induce transcription of their target genes, the upper limit for transcription factor induced transcription rates was set at 10^-4^/10^-4^=1 μM/μM/s.

#### Translation rates

For translation rates we use a similar logic as for the transcription rate: as upper limit we take the case where translation initiation is not limiting and the translation speed is bound by the ribosome progression and how much space the ribosome occupies on the transcript. We assume that the peptide elongation rate is constant, and it has been estimated at 10 amino acids/s (Boehlke and Friesen, 1975) and 10.5 amino acids/s (Waldron C, Jund R, 1974). It has been reported that ribosomes can stack together along a transcript as closely as 27 nucleotides apart (Wolin and Walter, 1988). Together, this gives a translation rate of 1.2 proteins/transcript/s when initiation is not limiting and all ribosomes progress unimpeded over the transcript. To allow for some margin of error, such as faster elongation of the specific proteins studied here, we set the maximal translation rate to 10 proteins/transcript/s. The lower limit is set at 10^-4^ proteins/transcript/s.

#### Degradation rates

For degradation rates, we withheld the available studies for validation purposes and so these could not be used for setting a prior distribution. We set the prior distribution for degradation rates to a wide range: between 10^-5^ and 1 s^-1^ for both proteins and transcripts, corresponding to a half-life between 19 hours and 0.7 seconds.

#### Inhibition rates

Inhibition was modeled by a non-linear function containing two parameters: the steepness and the 50%-inhibition concentration. The steepness is allowed to vary between 0.1 and 100. For the 50%-inhibition concentration, we used the same prior as for protein concentrations: between 0.1 nM and 100 μM.

#### Measurement variances

The prior for the measurement variance was set to an exponential distribution with λ=0.5 for time course data and λ=1.0 for steady state data.

### Likelihood

Firstly, the time average of the concentration of a transcript was calculated as

$\bar{m}_{i}=\frac{1}{2t_{cell\_cycle}}\int_{0}^{2t_{cell\_cycle}} m_{i}(t)dt$,

where *t_cell_cycle_* is the duration of the cell cycle (4800 seconds). Two full cell cycles were used, as the start of the first cell cycle can be affected by the method used to synchronize the cells and this effect can be alleviated by including a second cell cycle. Beyond the second cell cycle the cells typically start to diverge and are no longer synchronized. The average concentration of proteins is calculated in the same way with *p_i_* instead of *m_i_*.

For relative time course data measured using synchronized cells relative to unsynchronized cells, we modeled the relative value by dividing the modeled concentration a time *t_n_* by the time average and taking the log:

$$x_{i}(t_{n})=\log_{2}(\frac{m_{i}(t_{n})}{\bar{m}_{i}})$$

The likelihood function for the relative time course data is then defined as

$P(y_{i,t_{n}}|x_{i}(t_{n}))=t(y_{i,t_{n}}|\mu=x_{i}(t_{n}),\sigma=\sigma_{i},\nu=3)$,

where *y_i,tn_* is the measurement of gene *i* at time *t_n_* and *σ_i_* is the measurement variance for gene *i*. A *t*-distribution with three degrees of freedom is used as error model as a means of robust inference. The model cannot precisely represent the trajectories and the t-distribution can better accommodate the outlying measurements with respect to the model trajectories than the normal distribution.

For the absolute concentration data, the likelihood function is defined as

for transcripts, where *y_i_* is the log10-transformed measurement of the concentration of transcript *i* and *σ_j_* is the measurement variance for all transcripts in dataset *j*. For proteins the equation is identical but with mi replaced by *p_i_*. The likelihood is specified on a log scale as it is sufficient if the model captures the right order of magnitude of the measurement, rather than the precise concentration.

### Model checking

The model fit was investigated using the posterior predictive distribution and coefficients of determination.

The posterior predictive distribution is the probability distribution of a new set of data, given the model and the observed data. This distribution was approximated with the posterior Monte Carlo samples:

,

where *Θ*_1_*_..N_* are the Monte Carlo samples from the posterior distribution with each *Θ* being a vector containing all model parameters, and indicates the model.

The coefficients of determination for the time course data were calculated as

$R^{2}=1-\frac{\sum_{j=1}^{M} \sum_{i=1}^{N_{j}} (y_{j,i}-x(t_{i}))^{2}}{\sum_{j=1}^{M} \sum_{i=1}^{N_{j}} (y_{j,i}-\bar{y}_{j})^{2}}$,

where *j* indexes the time course gene expression datasets, *i* indexes the time points within that dataset, *x* is the modeled value at time *t_i_*, *y_i,j_* is the data value and *ȳ_j_* the mean of the data in that experiment. This equation corresponds to a null model which has a separate mean for each experiment.

The reference *R*^2^ was calculated by fitting a cubic spline to the data with the smoothing parameter selected through cross-validation, and then setting *x*(*t_i_*) in equal to the resulting spline value at *t_i_*. The smoothing spline was fitted using the R function smooth.spline with default settings.

## Supplementary References

Boehlke,K.W. and Friesen,J.D. (1975) Cellular content of ribonucleic acid and protein in Saccharomyces cerevisiae as a function of exponential growth rate: calculation of the apparent peptide chain elongation rate. *J. Bacteriol.*, **121**, 429–33.

Futcher,B. *et al.* (1999) A sampling of the yeast proteome. *Mol. Cell. Biol.*, **19**, 7357–68.

Hereford,L.M. and Rosbash,M. (1977) Number and distribution of polyadenylated RNA sequences in yeast. *Cell*, **10**, 453–62.

Mason,P.B. and Struhl,K. (2005) Distinction and relationship between elongation rate and processivity of RNA polymerase II in vivo. *Mol. Cell*, **17**, 831–40.

Selby,C.P. *et al.* (1997) RNA polymerase II stalled at a thymine dimer: Footprint and effect on excision repair. *Nucleic Acids Res.*, **25**, 787–93.

Tyson,C.B. and Lord,P.G. (1979) Dependency of size of Saccharomyces cerevisiae cells on growth rate. *J. Bacteriol.*, **138**, 92–8.

Waldron C, Jund R,L.F. (1974) The elongation rate of proteins of different molecular weight classes in yeast. *FEBS Lett.*, **46**, 11–6.

Wolin,S.L. and Walter,P. (1988) Ribosome pausing and stacking during translation of a eukaryotic mRNA. *EMBO J.*, **7**, 3559–69.

## Supplementary Figure 1

Posterior 90% confidence intervals for all model parameters, as inferred using the absolute steady state data, relative time course data or both.


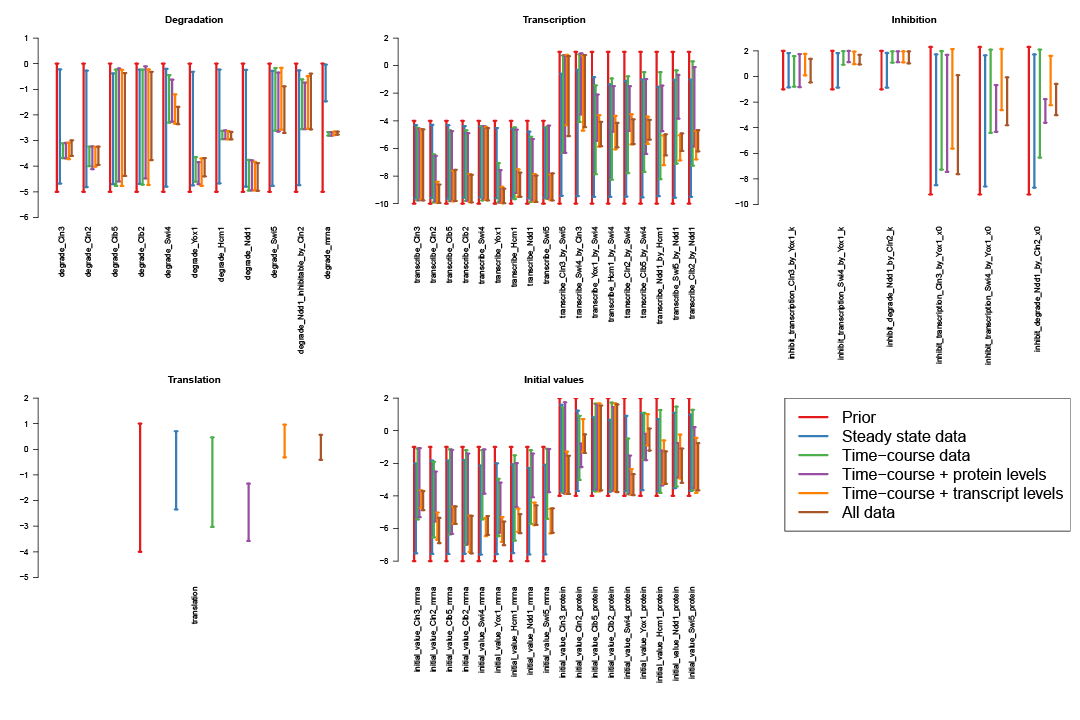


## Supplementary Figure 2

Marginal probability distribution density estimates for the parameters estimated from the different datasets. Bandwidths for the kernel density estimates were selected using Sheather-Jones bandwidth selection.


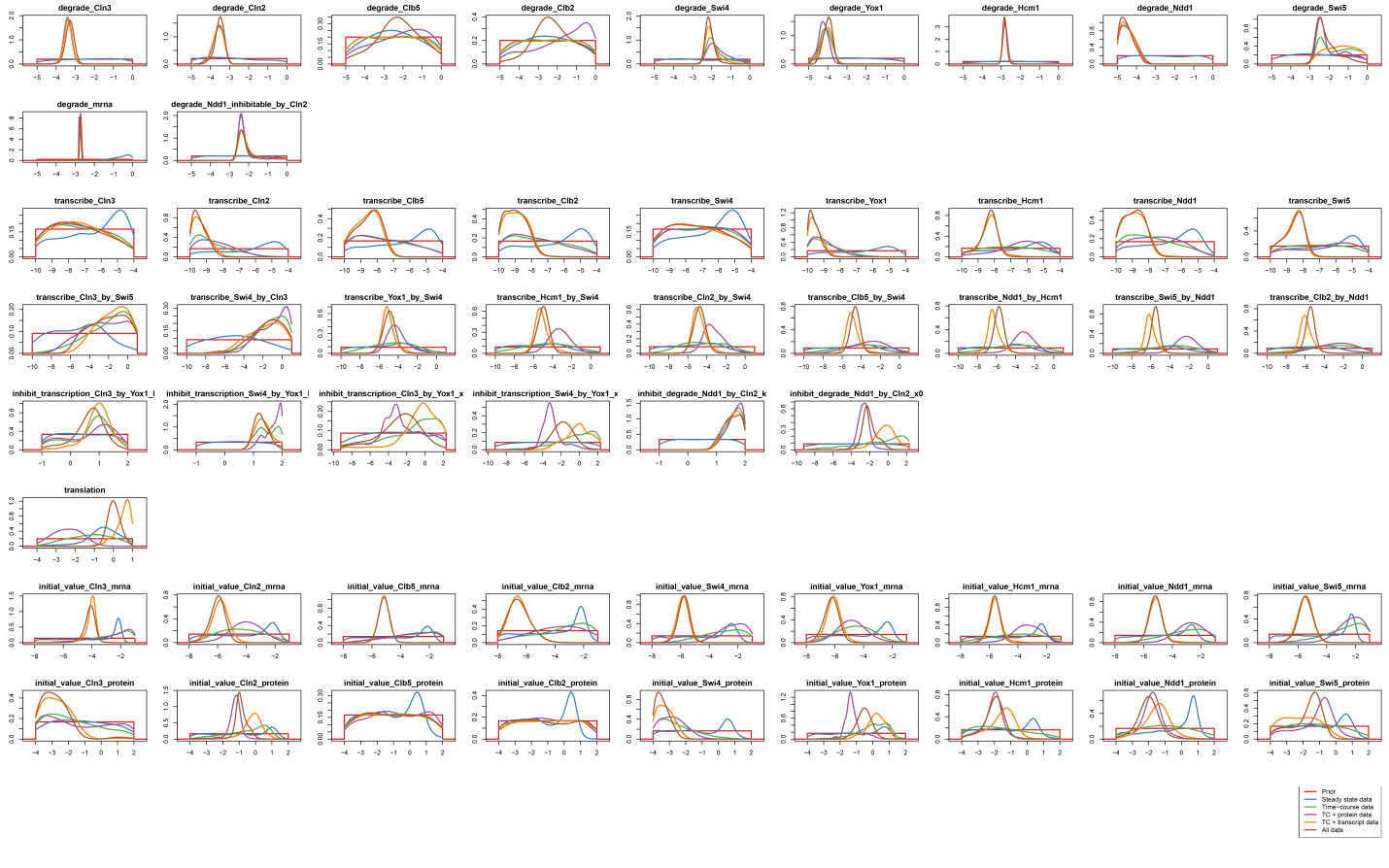


## Supplementary Table 1

|  | Prior | Steady state data | Time course data | Time course + protein | Time course + transcript | All data |
| --- | --- | --- | --- | --- | --- | --- |
| degrade_Cln3 | 5.00 | 4.45 | 0.58 | 0.60 | 0.64 | 0.61 |
| degrade_Cln2 | 5.00 | 4.54 | 0.76 | 0.89 | 0.77 | 0.71 |
| degrade_Clb5 | 5.00 | 4.32 | 4.54 | 4.41 | 4.53 | 4.02 |
| degrade_Clb2 | 5.00 | 4.45 | 4.49 | 4.37 | 4.51 | 3.44 |
| degrade_Swi4 | 5.00 | 4.60 | 1.86 | 1.64 | 1.14 | 0.68 |
| degrade_Yox1 | 5.00 | 4.43 | 0.96 | 0.86 | 1.07 | 0.71 |
| degrade_Hcm1 | 5.00 | 4.44 | 0.32 | 0.34 | 0.32 | 0.29 |
| degrade_Ndd1 | 5.00 | 4.55 | 1.19 | 1.18 | 1.12 | 1.08 |
| degrade_Swi5 | 5.00 | 4.49 | 2.44 | 2.30 | 2.41 | 1.82 |
| degrade_Ndd1_inhibitable_by_Cln2 | 5.00 | 4.48 | 1.94 | 1.81 | 2.05 | 2.18 |
| degrade_mrna | 5.00 | 1.43 | 0.14 | 0.14 | 0.14 | 0.12 |
| transcribe_Cln3 | 6.00 | 5.30 | 5.25 | 5.17 | 5.07 | 5.15 |
| transcribe_Cln2 | 6.00 | 5.27 | 3.47 | 3.31 | 1.52 | 1.33 |
| transcribe_Clb5 | 6.00 | 5.32 | 5.13 | 5.06 | 2.21 | 2.26 |
| transcribe_Clb2 | 6.00 | 5.18 | 5.12 | 4.91 | 2.03 | 1.99 |
| transcribe_Swi4 | 6.00 | 5.13 | 5.38 | 5.28 | 5.28 | 5.24 |
| transcribe_Yox1 | 6.00 | 5.08 | 2.83 | 2.35 | 1.18 | 1.05 |
| transcribe_Hcm1 | 6.00 | 5.04 | 5.19 | 4.58 | 1.99 | 1.75 |
| transcribe_Ndd1 | 6.00 | 4.84 | 4.72 | 4.41 | 2.01 | 1.91 |
| transcribe_Swi5 | 6.00 | 5.06 | 5.21 | 5.28 | 1.86 | 1.96 |
| transcribe_Cln3_by_Swi5 | 11.00 | 8.84 | 7.06 | 7.06 | 5.09 | 5.78 |
| transcribe_Swi4_by_Cln3 | 11.00 | 9.15 | 4.89 | 4.43 | 5.42 | 5.22 |
| transcribe_Yox1_by_Swi4 | 11.00 | 8.68 | 6.45 | 3.34 | 2.29 | 1.75 |
| transcribe_Hcm1_by_Swi4 | 11.00 | 8.15 | 7.34 | 3.29 | 2.43 | 1.76 |
| transcribe_Cln2_by_Swi4 | 11.00 | 8.37 | 7.01 | 3.23 | 2.19 | 1.78 |
| transcribe_Clb5_by_Swi4 | 11.00 | 8.52 | 7.23 | 5.42 | 1.98 | 1.43 |
| transcribe_Ndd1_by_Hcm1 | 11.00 | 7.91 | 7.74 | 3.30 | 2.14 | 1.52 |
| transcribe_Swi5_by_Ndd1 | 11.00 | 8.53 | 6.75 | 3.17 | 1.82 | 1.27 |
| transcribe_Clb2_by_Ndd1 | 11.00 | 8.49 | 7.56 | 5.75 | 2.08 | 1.54 |
| inhibit_transcription_Cln3_by_Yox1_k | 3.00 | 2.68 | 2.38 | 2.57 | 1.68 | 1.84 |
| inhibit_transcription_Swi4_by_Yox1_k | 3.00 | 2.69 | 1.06 | 0.87 | 0.98 | 0.75 |
| inhibit_transcription_Cln3_by_Yox1_x0 | 11.51 | 10.21 | 9.25 | 9.13 | 7.76 | 7.73 |
| inhibit_transcription_Swi4_by_Yox1_x0 | 11.51 | 10.24 | 6.49 | 3.66 | 4.77 | 3.74 |
| inhibit_degrade_Ndd1_by_Cln2_k | 3.00 | 2.71 | 0.89 | 0.85 | 0.86 | 0.93 |
| inhibit_degrade_Ndd1_by_Cln2_x0 | 11.51 | 10.39 | 8.42 | 1.84 | 3.85 | 2.43 |
| translation | 5.00 | 3.05 | 3.49 | 2.24 | 1.27 | 0.97 |
| initial_value_Cln3_mrna | 7.00 | 5.50 | 4.34 | 4.23 | 1.07 | 1.18 |
| initial_value_Cln2_mrna | 7.00 | 5.72 | 4.64 | 3.06 | 1.68 | 1.54 |
| initial_value_Clb5_mrna | 7.00 | 5.72 | 5.21 | 5.16 | 1.04 | 1.07 |
| initial_value_Clb2_mrna | 7.00 | 5.75 | 5.80 | 5.55 | 2.23 | 2.31 |
| initial_value_Swi4_mrna | 7.00 | 5.45 | 4.24 | 2.71 | 1.11 | 1.15 |
| initial_value_Yox1_mrna | 7.00 | 5.56 | 3.52 | 3.06 | 1.52 | 1.45 |
| initial_value_Hcm1_mrna | 7.00 | 5.44 | 5.23 | 2.68 | 1.42 | 1.17 |
| initial_value_Ndd1_mrna | 7.00 | 5.30 | 4.51 | 2.68 | 1.38 | 1.20 |
| initial_value_Swi5_mrna | 7.00 | 5.49 | 4.29 | 2.64 | 1.49 | 1.50 |
| initial_value_Cln3_protein | 6.00 | 5.29 | 5.25 | 5.60 | 2.60 | 2.34 |
| initial_value_Cln2_protein | 6.00 | 4.92 | 3.93 | 1.43 | 2.00 | 1.14 |
| initial_value_Clb5_protein | 6.00 | 4.51 | 5.37 | 5.22 | 5.39 | 5.20 |
| initial_value_Clb2_protein | 6.00 | 4.41 | 5.38 | 5.13 | 5.38 | 5.37 |
| initial_value_Swi4_protein | 6.00 | 4.62 | 3.41 | 2.36 | 1.58 | 1.27 |
| initial_value_Yox1_protein | 6.00 | 4.72 | 2.88 | 1.60 | 1.91 | 1.35 |
| initial_value_Hcm1_protein | 6.00 | 4.41 | 5.08 | 2.12 | 2.67 | 1.98 |
| initial_value_Ndd1_protein | 6.00 | 4.62 | 4.90 | 2.12 | 2.69 | 2.11 |
| initial_value_Swi5_protein | 6.00 | 4.67 | 4.94 | 3.74 | 3.38 | 2.89 |
